# Y772 phosphorylation of EphA2 is responsible for EphA2-dependent NPC nasopharyngeal carcinoma growth by Shp2/Erk-1/2 signaling pathway

**DOI:** 10.1038/s41419-020-02831-0

**Published:** 2020-08-27

**Authors:** Yi-Ping Xiang, Ta Xiao, Qi-Guang Li, Shan-Shan Lu, Wei Zhu, Yun-Ya Liu, Jie-Ya Qiu, Zheng-Hui Song, Wei Huang, Hong Yi, Yao-Yun Tang, Zhi-Qiang Xiao

**Affiliations:** 1 Department of Otolaryngology Head and Neck Surgery, Xiangya Hospital, Central South University, Changsha, 410008 China; 2 Research Center of Carcinogenesis and Targeted Therapy, Xiangya Hospital, Central South University, Changsha, 410008 China; 3 The Higher Educational Key Laboratory for Cancer Proteomics and Translational Medicine of Hunan Province, Xiangya Hospital, Central South University, Changsha, 410008 China; 4 Institute of Dermatology, Chinese Academy of Medical Sciences and Peking Union Medical College, Nanjing, 210042 China

**Keywords:** Head and neck cancer, Oncogenesis

## Abstract

EphA2 is an important oncogenic protein and emerging drug target, but the oncogenic role and mechanism of ligand-independent phosphorylation of EphA2 at tyrosine 772 (pY772-EphA2) is unclear. In this study, we established nasopharyngeal carcinoma (NPC) cell lines with stable expression of exogenous EphA2 and EphA2-Y772A (phosphorylation inactivation) using endogenous EphA2-knockdown cells, and observed that pY772A EphA2 was responsible for EphA2-promoting NPC cell proliferation and anchorage-independent and in vivo growth in mice. Mechanistically, EphA2-Y772A mediated EphA2-activating Shp2/Erk-1/2 signaling pathway in the NPC cells, and Gab1 (Grb2-associated binder 1) and Grb2 (growth factor receptor-bound protein 2) were involved in pY772-EphA2 activating this signaling pathway. Our results further showed that Shp2/Erk-1/2 signaling mediated pY772-EphA2-promoting NPC cell proliferation and anchorage-independent growth. Moreover, we observed that EphA2 tyrosine kinase inhibitor ALW-II-41-27 inhibited pY772-EphA2 and EphA2-Y772A decreased the inhibitory effect of ALW-II-41-27 on NPC cell proliferation. Collectively, our results demonstrate that pY772-EphA2 is responsible for EphA2-dependent NPC cell growth in vitro and in vivo by activating Shp2/Erk-1/2 signaling pathway, and is a pharmacologic target of ALW-II-41-27, suggesting that pY772-EphA2 can serve as a therapeutic target in NPC and perhaps in other cancers.

## Introduction

Nasopharyngeal carcinoma (NPC) is a head and neck cancer that shows a distinct endemic distribution with a high prevalence in Southern China and Southeast Asia, and remains one of the leading lethal malignancies in these areas^1^. Radiotherapy is the major therapeutic modality used to treat NPC and NPC patients can be cured if the disease is diagnosed and treated at an early stage. However, most of NPC patients are diagnosed at advanced stages due to nonspecific symptoms and these patients respond poorly to radiotherapy, whose prognosis is dismal^2^. Therefore, it is imperative to elucidate the underlying mechanism of development and progression of NPC, which could offer novel therapeutic targets.

EphA2 is a member of the EphA family of receptor tyrosine kinases (RTKs) and possesses both tumor-promoting role in a ligand-independent manner and tumor -suppressing role in a ligand-dependent manner^3,4^. EphA2 signals from both the receptor (forward signaling) and the ligand (reverse signaling) to form a communication system with critical and diverse roles in health and disease^3,4^. EphA2 activation by a ligand follows a classical pattern of RTK activation. When ligand Ephrin-A1 binds to EphA2, EphA2 occurs oligomer clustering and subsequent trans- and auto-phosphorylation of tyrosine residues in the juxtamembrane region and kinase domain^5^. This ligand- and tyrosine kinase-dependent EphA2 activation (reverse signaling) inhibits cancer cell proliferation, adhesion, and motility and tumor angiogenesis^6–9^. In contrast, ligand- and tyrosine kinase-independent EphA2 activation (forward signaling) promotes cancer development and progression^4,10,11^. Previous studies have shown that Serine897 (S897) and Tyrosine 772 (Y772) of EphA2 are important phosphorylated residues^4,6^. Although numerous studies have demonstrated that ligand-independent S897 phosphorylation of EphA2 (pS897-EphA2) promotes cancer development and progression^4,10–15^, the role of ligand-independent Y772 phosphorylation of EphA2 (pY772-EphA2) in cancers is unclear.

We recently used immunoprecipitation and mass spectrometry analysis (IP-MS) to search proteins interacted with EphA2 in NPC cells and found that Shp2, a protein tyrosine phosphatase (PTP), is an interactor of EphA2. PTPs play a crucial role in cancers^16^. Shp2 encoded by *PTPN11* (PTP, non-receptor type 11) gene is the first PTP to be identified as an oncogene^17,18^ and possesses an oncogenic role in the melanoma, leukemia, and lung and breast cancers^19–22^. Shp2 is implicated in the transduction of mitogenic, pro-survival, and pro-migratory signals from growth factor receptors^23^, and is required for the activation of Erk-1/2 signaling downstream of most RTKs^24–26^. EphA2 overexpression contributes to ErK-1/2 activation and cancer progression has been reported in many types of cancers^27,28^. A recent study indicates that EphA2 phosphorylates Shp2 and subsequently activates Erk-1/2^29^. However, whether ligand-independent pY772-EphA2 mediates EphA2-activating Shp2/Erk-1/2 signaling is unknown.

An ATP-competitive EphA2 tyrosine kinase inhibitor, ALW-II-41-27^30^, possesses obvious in vitro and in vivo anti-tumor effects in lung cancer^31–33^, melanoma^34^, triple-negative breast cancer^35^, and intrahepatic cholangiocarcinoma^36^. As an EphA2 tyrosine kinase inhibitor, whether ALW-II-41-27 inhibits cancer progression by inhibiting pY772-EphA2 has not been explored.

In the present study, we try to determine whether and how ligand-independent pY772-EphA2 promotes NPC growth, and tested whether pY772-EphA2 is a target of ALW-II-41-27. Our results demonstrate that pY772-EphA2 is responsible for EphA2-dependent NPC cell growth both in vitro and in vivo by activating the Shp2/Erk-1/2 signaling pathway, and that pY772-EphA2 is a pharmacologic target of ALW-II-41-27.

## Results

### pY772-EphA2 is responsible for EphA2-dependent NPC cell proliferation in vitro

**Fig. 1:**
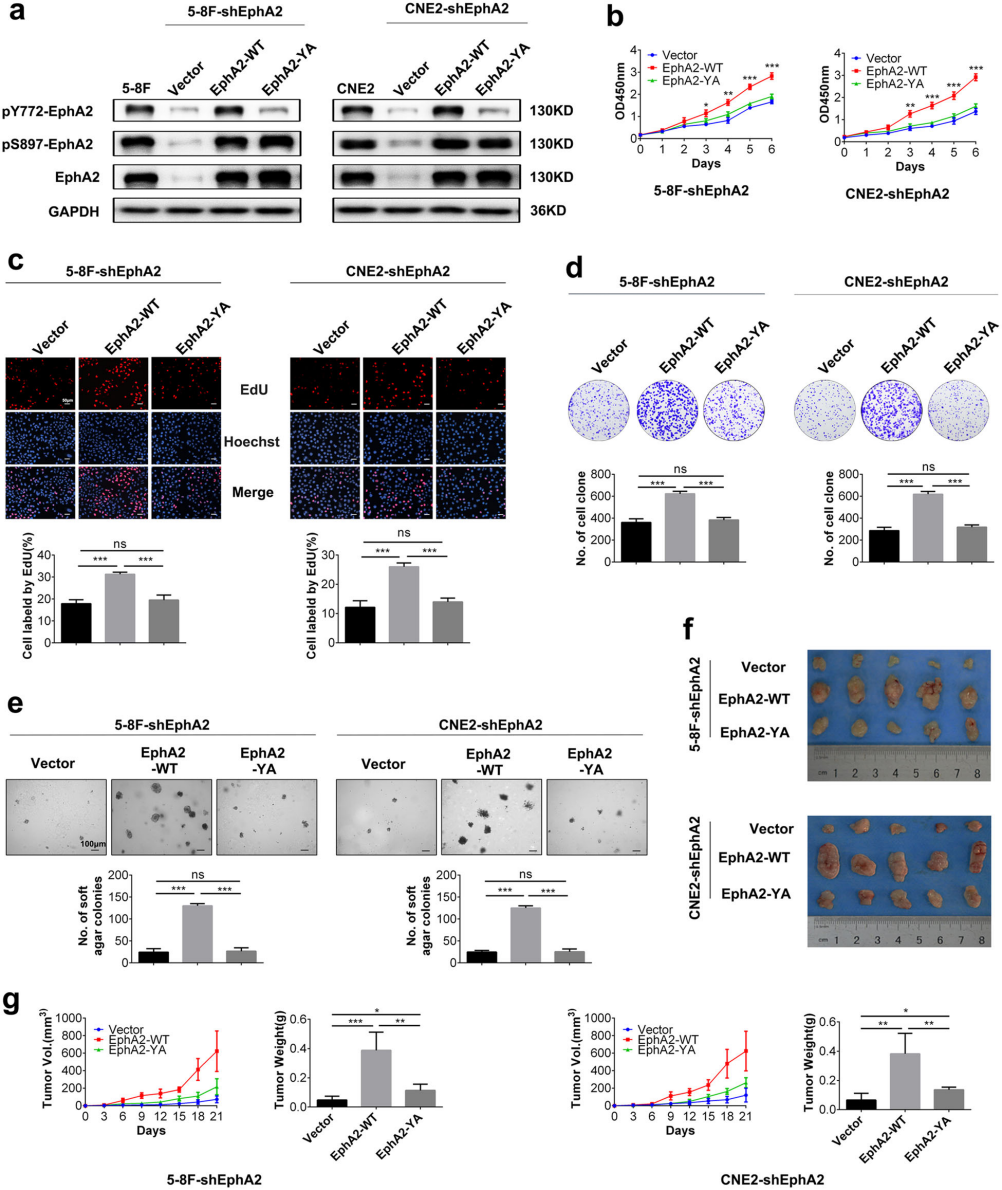
pY772-EphA2 is responsible for EphA2-dependent NPC cells growth in vitro and in vivo. **a** Establishment of 5-8F and CNE2 cell lines with the stable expression of exogenous EphA2 (EphA2-WT) or EphA2-Y772A (EphA2-YA) using endogenous EphA2-knockdown (shEphA2) cells. CCK-8 (**b**), EdU incorporation (**c**), and plate clone formation (**d**) assay showing the proliferation of NPC cells expressing EphA2-WT or EphA2-YA and their control cells. **e** Soft agar colony formation assay showing the anchorage-independent growth of NPC cells expressing EphA2-WT or EphA2-YA and their control cells. **f**, **g** Subcutaneous tumor formation experiment showing the growth of NPC cells expressing EphA2-WT or EphA2-YA and their control cells. The images of xenografts after 21 days subcutaneous implantation of the cells (**f**). Growth and weight of the xenograft tumors (**g**). *n* = 5 mice per group. Numbers represent mean ± SD. *P < 0.05; **P < 0.01; ***P < 0.001; n.s., no significance.

We previously established 5-8F and CNE2 NPC cell lines with stable knockdown of endogenous EphA2 by short hairpin RNA (shRNA) targeting EphA2 mRNA 3′-untranslated region, which were named as 5-8F-shEphA2 and CNE2-shEphA2, respectively^37^. To explore the functions of pY772-EphA2, we transfected plasmid expressing shRNA-resistant cDNA encoding EphA2 or EphA2-Y772A into 5-8F-shEphA2 and CNE2-shEphA2 cells, respectively, and established 5-8F and CNE2 cell lines with stable expression of exogenous EphA2 (EphA2-WT) or EphA2-Y772A (EphA2-YA). Western blotting showed that the established 5-8F and CNE2 cell lines expressed the equivalent levels of exogenous EphA2-WT and EphA2-YA, and Y772A mutation abolished the phosphorylation of EphA2 at Y772 (pY772-EphA2) but did not affect the phosphorylation of EphA2 at S897 (pS897-EphA2) (Fig. 1a). Next, we analyzed the effects of EphA2-WT and EphA2-YA on the NPC cell proliferation. Cell counting kit-8 (CCK-8), plate colony formation, and 5-ethynyl-2′-deoxyuridine (EdU) incorporation labeling assay showed that EphA2-WT dramatically increased NPC cells proliferation in vitro, whereas EphA2-YA failed to do it as compared to endogenous EphA2 knockdown (Fig. 1b–d), indicating that Y772A mutation abolished the effects of EphA2-WT on NPC cell proliferation in vitro. Together, these results demonstrate that pY772-EphA2 is responsible for EphA2-dependent NPC cells proliferation in vitro.

### pY772-EphA2 is responsible for EphA2-dependent anchorage-independent growth and in vivo tumorigenicity of NPC cells

Soft agar colony formation assay showed that EphA2-WT dramatically increased the anchorage-independent growth of NPC cells, whereas EphA2-YA failed to do it as compared to endogenous EphA2 knockdown (Fig. 1e), indicating that Y772A mutation abolished the effects of EphA2-WT on the anchorage-independent growth of NPC cells. Tumor formation experiment showed that EphA2-WT dramatically increased in vivo tumorigenicity of NPC cells, whereas EphA2-YA slightly did it as compared to endogenous EphA2 knockdown (Fig. 1f, g), indicating that Y772A mutation almost abolished the effects of EphA2-WT on the in vivo tumorigenicity of NPC cells. Together, these results demonstrate that pY772-EphA2 is responsible for EphA2-dependent anchorage-independent growth and in vivo tumorigenicity of NPC cells.

### Identification of Shp2 as a protein that interacted with EphA2 in the NPC cells

**Fig. 2:**
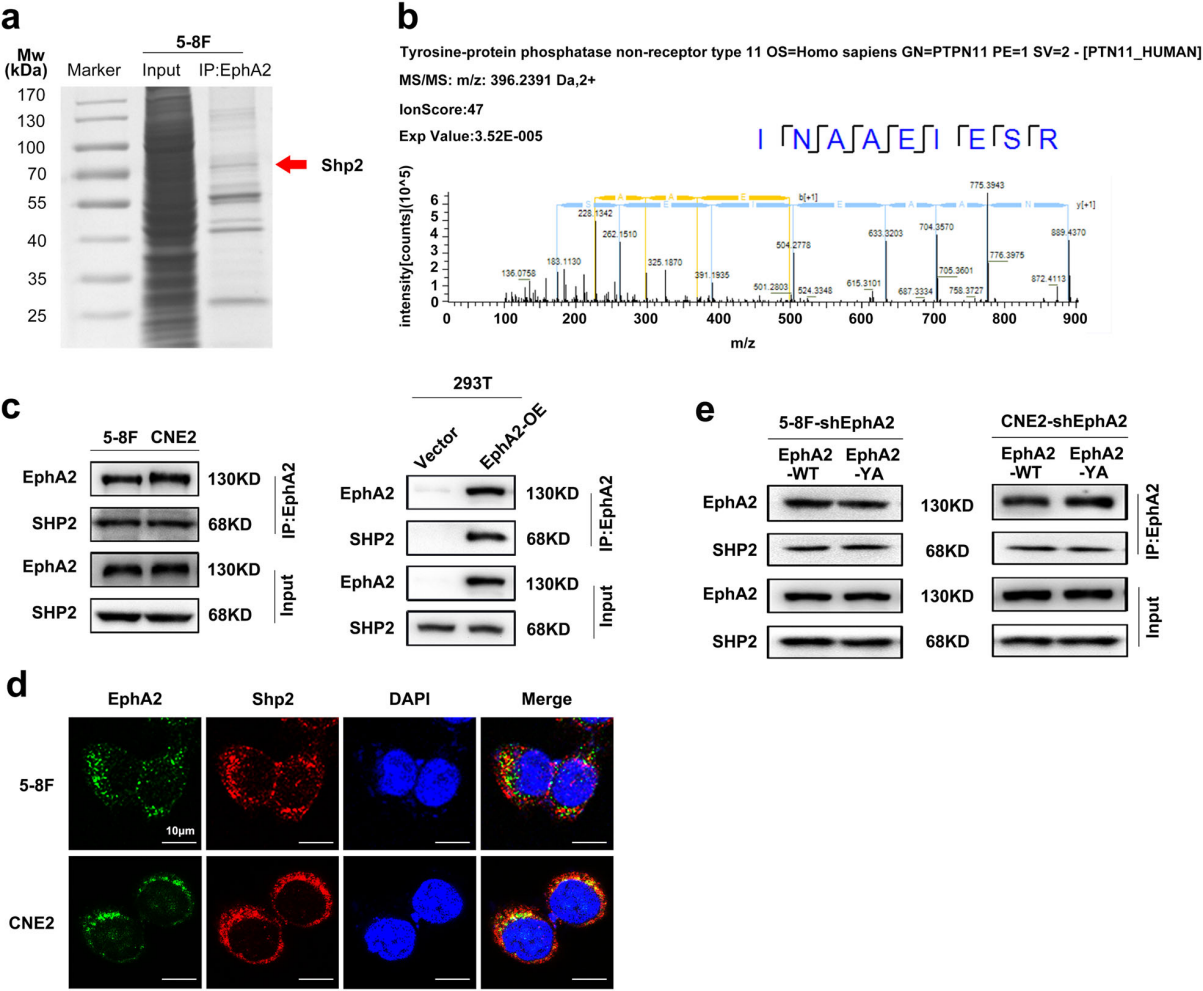
Shp2 is a protein that interacted with EphA2 in the NPC cells. **a** IP-mass spectrometry (MS) analysis of the proteins interacted with EphA2. Total cell proteins from 5-8F NPC cells were subjected to co-immunoprecipitation with anti-EphA2 antibody. The coimmunoprecipitated protein complex was resolved on SDS-PAGE and Coomassie blue staining, and then the bands were retrieved and analyzed by MS analysis. **b** Identification of Shp2 as a protein that interacted with EphA2 by MS analysis. The amino acid sequence of a doubly charged peptide with *m*/*z* 396.2391 Da was identified as NAAEIESR and Mascot search showing the peptide matched with Shp2. **c** Co-IP confirming the interaction of Shp2 and EphA2. Total cell proteins from the 5-8F and CNE2 NPC cells (left) and HEK293 cells ectopically expressing EphA2 (right) were prepared and subjected to immunoprecipitation (IP) with anti-EphA2 antibody followed by immunoblotting with antibodies against Shp2 or EphA2. **d** Immunofluorescence showing the colocalization of EphA2 (green) and Shp2 (red) in the NPC cells. 5-8F and CNE2 cells were incubated with mouse anti-EphA2 and rabbit anti-Shp2 antibodies followed by staining with DyLight® 488 anti-mouse IgG and DyLight® 594 anti-rabbit IgG, and observed by confocal fluorescence microscopy. Scale bar = 10 μm. **e** Co-IP showing that EphA2-Y772A mutation does not disturb the interaction of EphA2 and Shp2 in the NPC cells. Total cell proteins from the 5-8F and CNE2 cells expressing EphA2-WT or EphA2-YA were prepared and subjected to immunoprecipitation (IP) with anti-EphA2 antibody followed by immunoblotting with antibodies against Shp2 or EphA2.

To search proteins that interact with EphA2, EphA2 interactors were coimmunoprecipitated with anti-EphA2 antibody from NPC cell extracts, separated on SDS-polyacrylamide gel electrophoresis (PAGE), and stained with Coomassie blue (Fig. 2a). All protein bands were excised and subjected to liquid chromatography and high-throughput MS (LC-MS/MS) analysis, the proteomic data of which are available via ProteomeXchange with identifier PXD015242. Shp2, identified as an interactor of EphA2 (Fig. 2b), was selected for further investigation. Co-IP confirmed that Shp2 interacted with EphA2 in the two NPC cell lines (Fig. 2c). Shp2 was coimmunoprecipitated with exogenous EphA2 from the extracts of HEK293 cells transfected with EphA2 expression plasmid (Fig. 2c). Immunofluorescent staining showed that Shp2 and EphA2 were colocalized in the NPC cells (Fig. 2d). Moreover, co-IP showed that Y772A mutation did not disturb the interaction of EphA2 and Shp2 in the NPC cells, indicating that interaction of EphA2 and Shp2 is not dependent on pY772-EphA2 (Fig. 2e). Collectively, these data provide evidence that Shp2 interacts with EphA2 in the NPC cells.

### pY772-EphA2 mediates EphA2-activating Shp2/Erk-1/2 signaling pathway in NPC cells

**Fig. 3:**
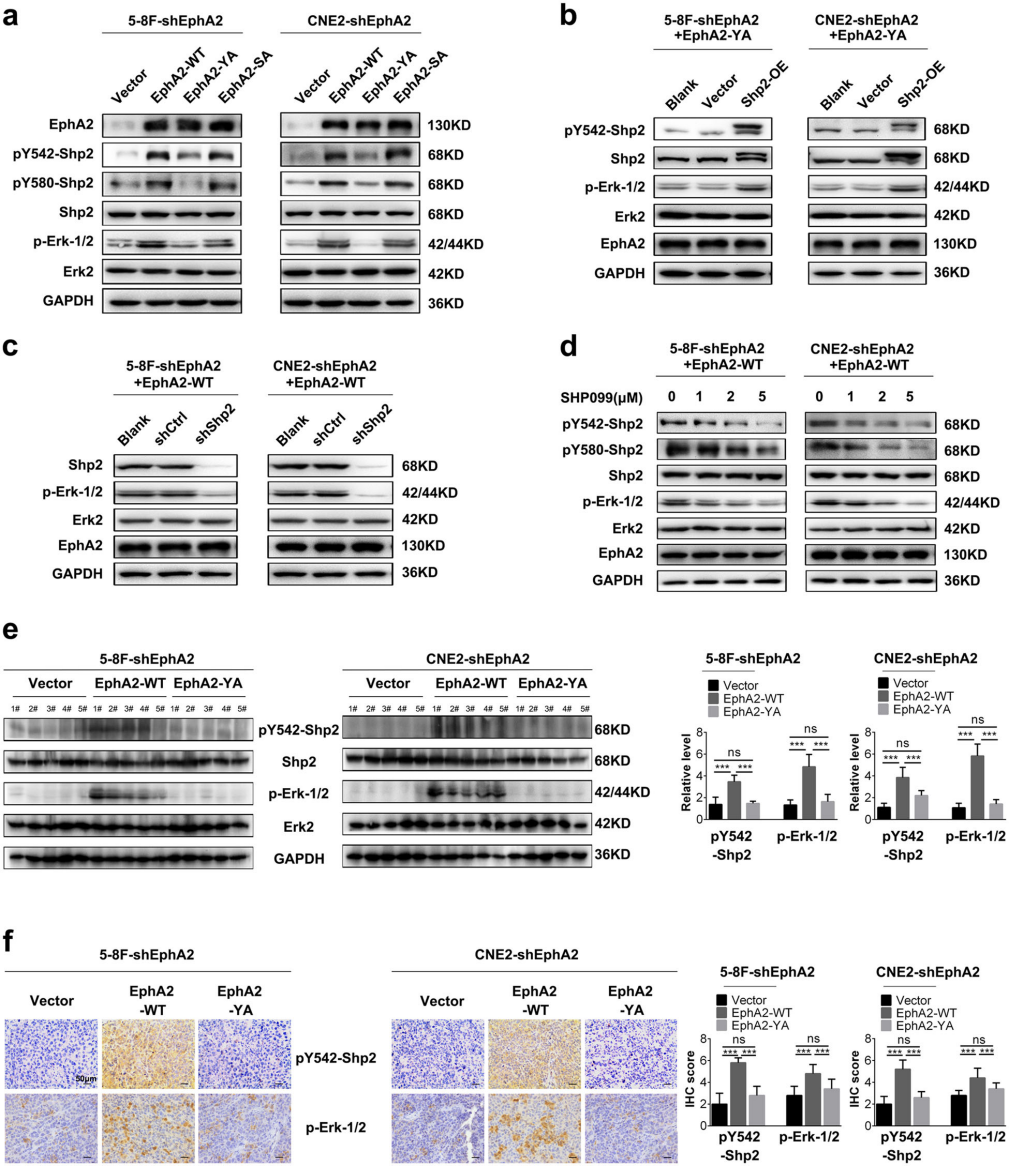
pY772-EphA2 mediates EphA2-activating Shp2/Erk-1/2 signaling pathway in the NPC cells. **a** Western blotting showing the levels of p-Shp2 and p-Erk-1/2 in the NPC cells expressing EphA2-WT, EphA2-YA, or EphA2-SA and their control cells. **b** Western blotting showing the levels of p-Erk-1/2 in the EphA2-YA-expressed NPC cells transfected with Shp2 expression plasmid and their control cells. **c** Western blotting showing the levels of p-Erk-1/2 in the EphA2-WT-expressed NPC cells infected with the lentiviral vector expressing Shp2 shRNA. **d** Western blotting showing the levels of p-Erk-1/2 in the EphA2-WT-expressed NPC cells treated with Shp2 inhibitor SHP099 for 12 h. **e** Western blotting showing the levels of p-Shp2 and p-Erk-1/2 in the xenografts of NPC cells expressing EphA2-WT or EphA2-YA and their control cells. **f** Immunohistochemistry (IHC) showing the levels of p-Shp2 and p-Erk-1/2 in the xenografts of NPC cells expressing EphA2-WT or EphA2-YA and their control cells. Representative IHC images are shown on the left and statistical analysis is presented on the right. Scale bar = 50 μm. Numbers represent mean ± SD. ***P < 0.001; no, no significance. OE overexpression; shCtrl, scramble non-target RNA.

Shp2 acts as a positive regulator of Erk-1/2 pathway and is required for the activation of Erk-1/2 signaling downstream of most RTKs^24–26^. The recent study has indicated that EphA2 activates Erk-1/2 by phosphorylating Y542/580 of Shp2^29^. Our results showed that Shp2 interacted with EphA2 in the NPC cells (Fig. 2). Therefore, we detected whether pY772-EphA2 mediates EphA2-activating Shp2/ErK-1/2 signaling pathway in the NPC cells and observed that EphA2-WT significantly increased while EphA2-YA failed to increase the levels of p-Shp2 and p-Erk-1/2, as compared to endogenous EphA2 knockdown (Fig. 3a), indicating that Y772A mutation abolished the effect of EphA2-WT on the phosphorylated levels of Shp2 and Erk-1/2. Moreover, we observed that S897A mutation (EphA2-SA) did not affect the levels of p-Shp2 and p-Erk-1/2 (Fig. 3a).

Previous studies have demonstrated that EphA2 interacts with and phosphorylates Shp2, then phosphorylated Shp2 activates downstream Erk-1/2^29^. Therefore we investigated whether pY772-EphA2 phosphorylates and activates Erk-1/2 via Shp2 in the NPC cells and observed that Shp2 overexpression restored p-Erk-1/2 levels in the NPC cells expressing EphA2-YA (Fig. 3b); either Shp2 knockdown or Shp2 inhibitor SHP099 dramatically decreased p-Erk-1/2 levels in the NPC cells expressing EphA2-WT (Fig. 3c, d). Moreover, western blotting and immunohistochemistry (IHC) showed that EphA2-WT increased the levels of p-Shp2 and p-Erk-1/2 in the xenografts of NPC cells, whereas EphA2-YA failed to do it as compared to endogenous EphA2 knockdown (Fig. 3e, f). Together, the results indicate that pY772-EphA2 mediates EphA2-activating Shp2/Erk-1/2 signaling pathway in the NPC cells.

### pY772-EphA2 promotes NPC cell proliferation by Shp2/Erk-1/2 signaling pathway

**Fig. 4:**
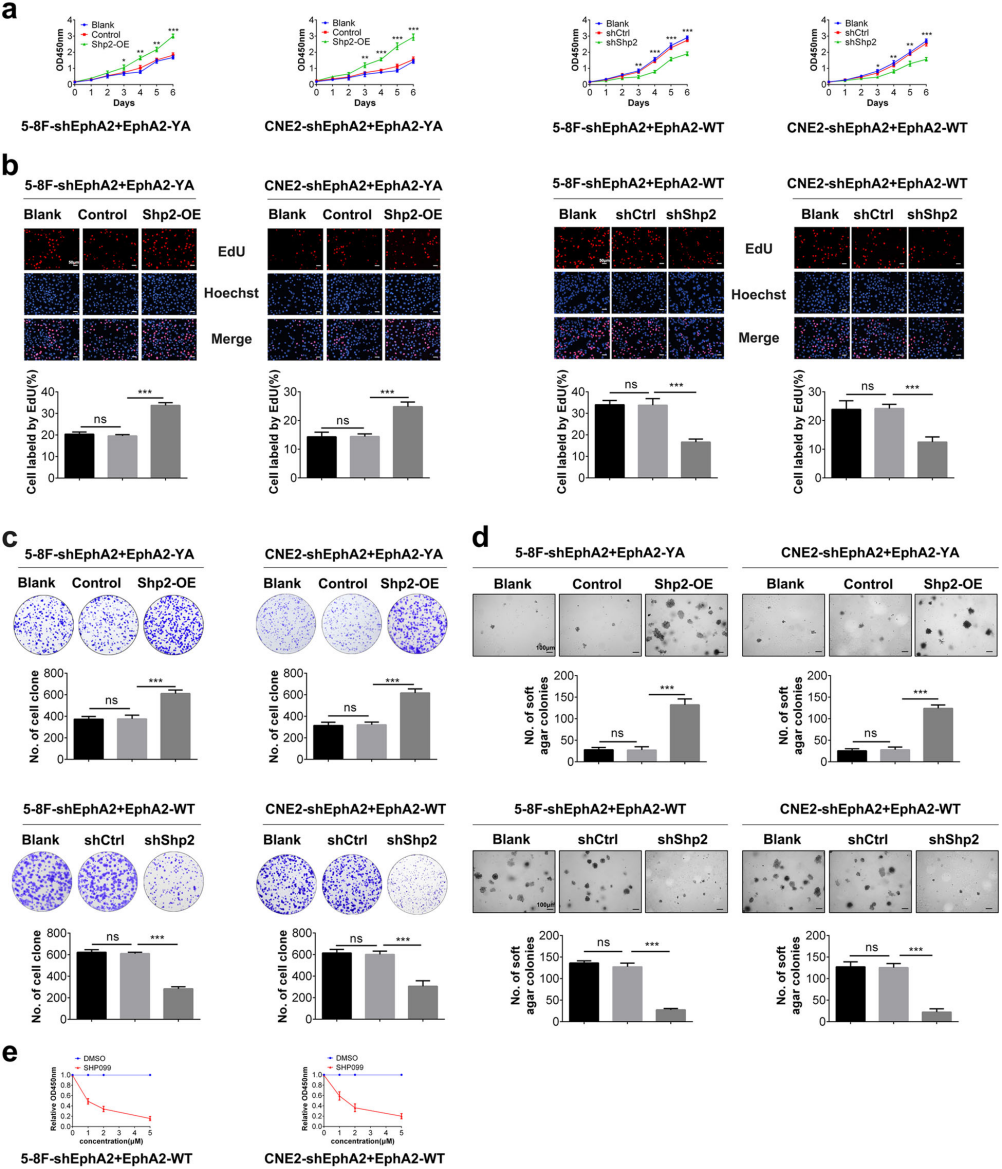
pY772-EphA2 promotes NPC cell proliferation and anchorage-independent growth by Shp2 signaling. CCK-8 (**a**), EdU incorporation (**b**), and plate clone formation (**c**) assay showing the proliferation of EphA2-YA-expressed NPC cells transfected with Shp2 expression plasmid, EphA2-WT-expressed NPC cells infected with the lentiviral vector expressing Shp2 shRNA, and their control cells. **d** Soft agar colony formation assay showing the anchorage-independent growth of EphA2-YA-expressed NPC cells transfected with Shp2 expression plasmid, EphA2-WT-expressed NPC cells infected with the lentiviral vector expressing Shp2 shRNA, and their control cells. **e** Shp2 inhibitor SHP099 inhibits EphA2-WT-expressed NPC cell proliferation. Cells were treated with 1, 2, or 5 μm SHP099 for 72 h and cell proliferation was assayed by CCK-8. Cell proliferation in SHP099 treatment group relative to a DMSO control group is shown. Numbers represent mean ± SD. *P < 0.05; **P < 0.01; ***P < 0.001; n.s., no significance.

**Fig. 5:**
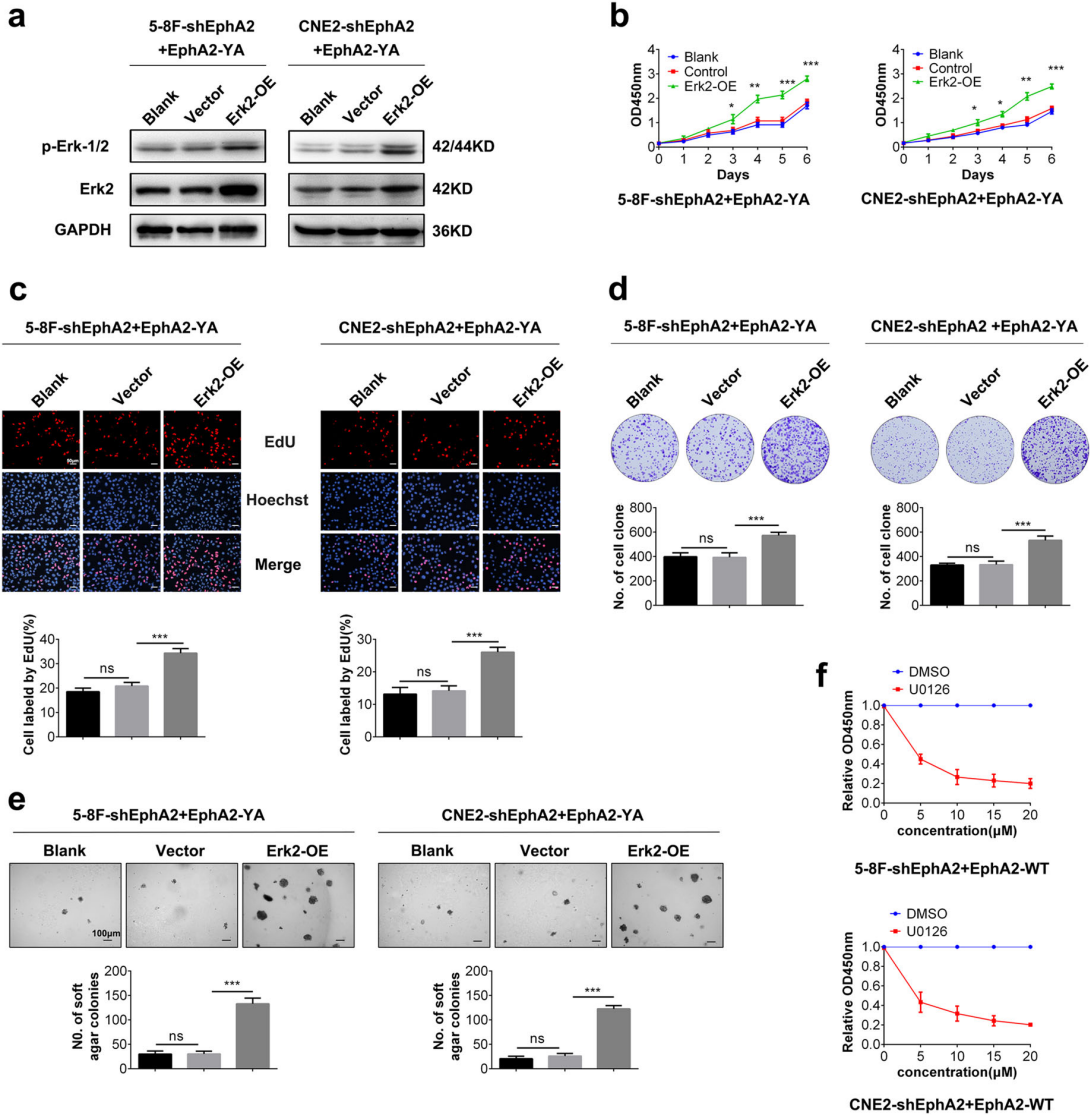
pY772-EphA2 promotes NPC cell proliferation by Erk-1/2 signaling. **a** Western blotting showing the levels of Erk-1/2 and p-Erk-1/2 in the EphA2-YA-expressed NPC cells transfected with Erk-2 expression plasmid, and their control cells. CCK-8 (**b**), EdU incorporation (**c**), and plate clone formation (**d**) assay showing the proliferation of EphA2-YA-expressed NPC cells transfectedwith Erk-2 expression plasmid, and their control cells. **e** Soft agar colony formation assay showing the anchorage-independent growth of EphA2-YA-expressed NPC cells transfected with Erk-2 expression plasmid, and their control cells. **f** MEK inhibitor U0126 inhibits EphA2-WT-expressed NPC cells proliferation. Cells were treated with 5–20 μm U0126 for 72 h and cell proliferation was assayed by CCK-8. Shown is cell proliferation in U0126 treatment group relative to a DMSO control group. Numbers represent mean ± SD. *P < 0.05; **P < 0.01; ***P < 0.001; n.s., no significance.

We next explore whether Shp2/Erk-1/2 signaling pathway mediates pY772-EphA2-promoting NPC cell proliferation. CCK-8, plate colony formation, and EdU incorporation labeling assay showed that Shp2 overexpression restored the proliferation of NPC cells expressing EphA2-YA and Shp2 knockdown dramatically decreased the proliferation of NPC cells expressing EphA2-WT (Fig. 4a–c). Soft agar colony formation assay showed that Shp2 overexpression restored the anchorage-independent growth of NPC cells expressing EphA2-YA and Shp2 knockdown dramatically decreased the anchorage-independent growth of NPC cells expressing EphA2-WT (Fig. 4d). CCK-8 assay also showed that SHP099 inhibited the proliferation of NPC cells expressing EphA2-WT in a dose-dependent manner (Fig. 4e). Moreover, CCK-8, plate colony formation, and EdU incorporation labeling assay showed that Erk-2 overexpression restored the proliferation and anchorage-independent growth of NPC cells expressing EphA2-YA (Fig. 5a–e) and CCK-8 assay showed that MEK inhibitor U0126 inhibited the proliferation of NPC cells expressing EphA2-WT in a dose-dependent manner (Fig. 5f). Collectively, the results demonstrate that Shp2/Erk-1/2 signaling pathway mediates pY772-EphA2-promoting NPC cell proliferation and anchorage-independent growth.

### pY772-EphA2 is a target of EphA2 tyrosine kinase inhibitor ALW-II-41-27 in the NPC cells

**Fig. 6:**
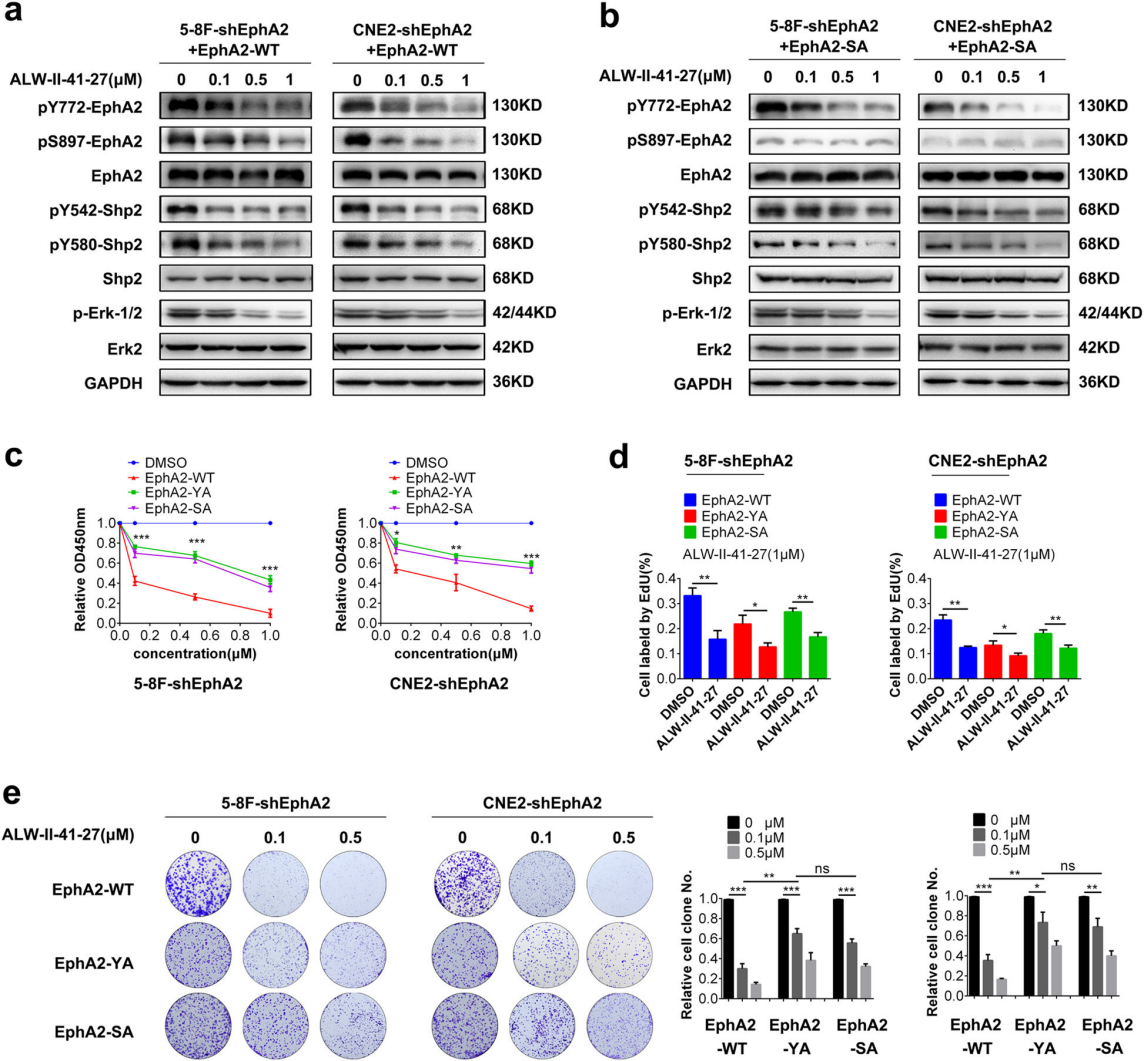
pY772-EphA2 is a target of EphA2 inhibitor ALW-II-41-27 in the NPC cells. **a** Western blotting showing the levels of pY772-EphA2, pS897-EphA2, p-Shp2 (Y542/Y580) and p-Erk-1/2 in the EphA2-WT-expressed NPC cells treated with ALW-II-41-27 for 6 h. **b** Western blotting showing the levels of pY772-EphA2, pS897-EphA2, p-Shp2 (Y542/Y580) and p-Erk-1/2 in the EphA2-SA (S897A)-expressed NPC cells treated with ALW-II-41-27 for 6 h. **c**–**e** ALW-II-41-27 inhibits EphA2-WT-expressed NPC cell proliferation. Cells were treated with ALW-II-41-27 and cell proliferation was assayed by CCK-8 (**c**), EdU incorporation (**d**), and plate clone formation (**e**). Numbers represent mean ± SD. *P < 0.05; **P < 0.01; ***P < 0.001; ns, no significance.

ALW-II-41-27, a novel EphA2 tyrosine kinase inhibitor^30^, has obvious anti-tumor effects in the many types of cancers^31–36^. Previous studies indicate that ALW-II-41-27 suppresses cancers by targeting S879 phosphorylation of EphA2^32,34,36^. As our results showed that pY772-EphA2 plays a role in NPC growth, we detected whether pY772-EphA2 is a target of ALW-II-41-27. Western blotting showed that ALW-II-41-27 reduced the levels of pY772-EphA2, pS897-EphA2, p-Shp2, and p-Erk-1/2 in the NPC cells expressing EphA2-WT in a dose-dependent manner (Fig. 6a), indicating that ALW-II-41-27 inhibits the activation of Y772-EphA2 and its downstream Shp2/Erk-1/2 signaling. We also observed that ALW-II-41-27 reduced the levels of pY772-EphA2, and p-Shp2 and p-Erk-1/2 in the NPC cells expressing EphA2-S897A (EphA2-SA) in a dose-dependent manner (Fig. 6b), indicating that ALW-II-41-27 inhibits the activation of Y772-EphA2 and its downstream Shp2/Erk-1/2 signaling in a pS897-EphA2-independent manner. Next, we analyzed the effect of ALW-II-41-27 on the proliferation of NPC cells expressing EphA2-WT, EphA2-YA, or EphA2-SA. CCK-8, EdU incorporation labeling, and plate colony formation assay showed that either EphA2-YA or EphA2-SA obviously decreased the inhibitory effect of ALW-II-41-27 on NPC cell proliferation as compared to EphA2-WT (Fig. 6c–e). Together, our results indicate that pY772-EphA2 is a pharmacologic target of ALW-II-41-27 in the NPC cells.

### Gab1 and Grb2 are involved in pY772-EphA2-activating Shp2/Erk-1/2 signaling pathway in NPC cells

**Fig. 7:**
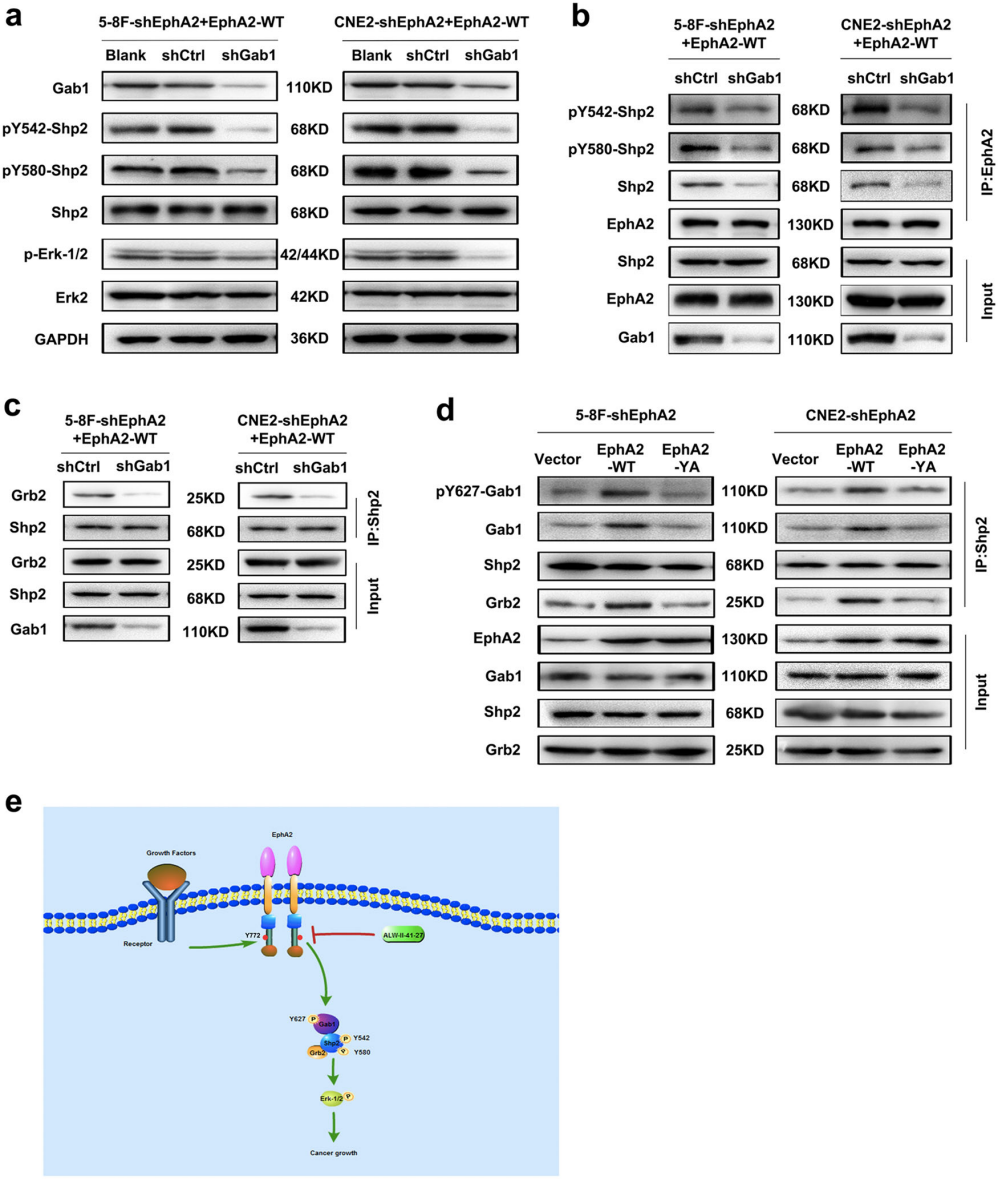
Gab1 and Grb2 are involved in pY772-EphA2-activating Shp2/Erk-1/2 signaling pathway in the NPC cells. **a** Western blotting showing the levels of p-Shp2 (Y542/Y580) and p-Erk-1/2 in the EphA2-WT-expressed NPC cells infected with the lentiviral vector expressing Gab1 shRNA, and their control cells. **b** Co-IP showing the levels of p-Shp2(Y542/580) and Shp2 bound to EphA2 in the EphA2-WT-expressed NPC cellsinfected with the lentiviral vector expressing Gab1 shRNA, and their control cells. Total cell proteins from the infected cells were prepared and subjected to immunoprecipitation immunoprecipitation (IP) with anti-EphA2 antibody followed by immunoblotting with antibodies against p-Shp2 (Y542/Y580), Shp2, or EphA2. **c** Co-IP analysis showing the levels of Grb2 bound to Shp2 in the EphA2-WT-expressed NPC cells infected with the lentiviral vector expressing Gab1 shRNA, and their control cells. Total cell proteins from the infected cells were prepared and subjected to immunoprecipitation (IP) with anti-Shp2 antibody followed by immunoblotting with antibodies against Grb2 or Shp2. **d** Co-IP showing the levels of p-Gab1, Gab1, and Grb2 bound to Shp2 in the NPC cells expressing EphA2-WT or EphA2-YA and their control cells. Total cell proteins from the indicated cells were prepared and subjected to immunoprecipitation with anti-Shp2 antibody followed by immunoblotting with antibodies against p-Gab1, Gab1, Grb2, or Shp2. **e** A model for pY772-EphA2-dependent NPC cell growth. EphA2 is phosphorylated at Y772 by growth factors in a ligand-independent manner and phosphorylation of EphA2 at Y772 (pY772-EphA2) activates Shp2/Erk-1/2 signaling, and both Gab1 and Grb2 are involved in pY772-EphA2-activating Shp2/Erk-1/2 activation, which promotes NPC cell growth. EphA2 tyrosine kinase inhibitor ALW-II-41-27 inhibits pY772-EphA2, which blocks NPC cell growth.

It has been reported that RTK activated by growth factors recruits and phosphorylates an adaptor scaffolding protein, Gab1, then phosphorylated Gab1 becomes associated with Shp2, and this association of Shp2 with Gab1 leads to Erk-1/2 activation^29,38^. In addition, it has been suggested that the phosphorylated Shp2 recruits Grb2 to activate Erk-1/2^29,38,39^. Therefore, we analyzed whether pY772-EphA2-activating Shp2/Erk-1/2 requires Gab1 and Grb2 in the NPC cells. The results showed that Gab1 knockdown by shRNA obviously decreased the phosphorylated levels of Shp2 and Erk-1/2 (Fig. 7a), and also reduced Shp2 and p-Shp2 bound to EphA2 (Fig. 7b) and Grb2 bound to Shp2 (Fig. 7c), indicating that Gab1 is required for EphA2-activating Shp2/Erk-1/2 signaling pathway possibly by increasing the association of Shp2 with EphA2 and Grb2. Moreover, we observed that EphA2-WT increased p-Gab1, Gab1, and Grb2 bound to Shp2, whereas EphA2-YA failed to do it as compared to endogenous EphA2 knockdown (Fig. 7d), indicating that Y772 mutation abolished EphA2-WT-promoting the association of Shp2 with p-Gab1 and Grb2. Collectively, Our results indicate that Gab1 and Grb2 are involved in pY772-EphA2-activating Shp2/Erk-1/2 in the NPC cells, and Gab1 is located in the upstream of Shp2 and Grb2 is located in the downstream of Shp2 (Fig. 7e).

## Discussion

EphA2 is an important oncogenic protein and emerging drug target^3,40,41^, but the ligand-independent oncogenic role and mechanism of pY772-EphA2 are unclear. To determine the role of pY772-EphA2 in NPC, we established NPC cell lines expressing the equal levels of exogenous EphA2-WT and EphA2-Y772A using endogenous EphA2 knockdown cells, an appropriate cell model for comparing the differences of biological functions in the EphA2-WT and EphA2-Y772A. Using the established cell lines, we observed that Y772A mutation abolished the effects of EphA2 on NPC cells growth in vitro and in vivo. Our results for the first time demonstrate that pY772-EphA2 is responsible for EphA2-dependent NPC cells growth in vitro and in vivo.

The signals downstream of pY772-EphA2 underlying its growth promotion are completely unclear. Our targeted proteomics analysis found that Shp2 interacts with EphA2 in the NPC cells. Previous studies have demonstrated that Shp2 is required for the activation of Erk-1/2 signaling downstream of most RTKs including EphA2^24–26,29^. Therefore, we determined whether pY772-EphA2 activates Shp2/Erk-1/2 signaling pathway in the NPC cells and demonstrate that pY772-EphA2 mediates EphA2-activating Shp2/Erk-1/2 signaling pathway in the NPC cells and xenografts. Moreover, it has been reported that EphA2 promotes cancer progression by pS897-EphA2-activating AKT/Stat3 signaling^4,12–15,37^. Therefore, we detected the effect of pY772-EphA2 on AKT/Stat3 signaling activity in the NPC cells and observed that EphA2-Y772A did not affect the phosphorylated levels of AKT and Stat3, whereas EphA2-S897A reduced the phosphorylated levels of AKT and Stat3 as compared to endogenous EphA2-WT (Supplementary Fig. 1), indicating that pY772-EphA2- and pS897-EphA2-activating oncogenic signaling pathways are different in the NPC cells.

Whether and how Gab1 and Grb2 are involved in EphA2-activating Shp2/Erk-1/2 signaling pathway in a ligand-independent manner are still unclear^29^. Our results showed that Gab1 is required for EphA2-activating Shp2/Erk-1/2 signaling pathway possibly by increasing the association of Shp2 with EphA2 and Grb2, and Y772 mutation abolished EphA2-WT-promoting the association of Shp2 with p-Gab1 and Grb2. The results indicate that Gab1 and Grb2 are involved in pY772-EphA2-activating Shp2/Erk-1/2 signaling pathway in the NPC cells.

Next, we determined whether Shp2/Erk-1/2 signaling pathway mediates pY772-EphA2-promoting NPC cell proliferation and anchorage-independent growth. Using lose and gain of function approaches, we demonstrate that Shp2/Erk-1/2 signaling mediates pY772-EphA2-promoting NPC cell proliferation and anchorage-independent growth. Our data suggest that pY772-EphA2/Shp2/Erk-1/2 signaling axis has an important oncogenic function, highlighting the potential of this signaling axis for treating NPC.

EphA2 is an emerging drug target in cancers^40,41^. Various therapeutic strategies targeting EphA2, such as EphA2 antibody, ligand Ephrin-A1, Ephrin-A1 mimic peptides, and RNA interference, have been developed^41^. It has been reported that EphA2 tyrosine kinase inhibitor, ALW-II-41-27^30^, has obvious anti-tumor effects in the many types of cancers^31–36^. As an EphA2 tyrosine kinase inhibitor, whether ALW-II-41-27 suppresses cancers by inhibiting pY772-EphA2 is unclear. We observed that ALW-II-41-27 inhibited the levels of pY772-EphA2 and its downstream p-Shp2 and p-Erk-1/2 in the NPC cells in a pS897-EphA2-independent manner. Functionally, EphA2-Y772A obviously decreased the inhibitory effect of ALW-II-41-27 on NPC cell proliferation. Our data suggest that pY772-EphA2 is a target of ALW-II-41-27 in cancer cells.

The last question is what phosphorylates EphA2 at Y772 in the NPC cells. It has been reported that fetal bovine serum (FBS) induces pS897-EphA2 in cancer cells by a ligand-independent mechanism^4,13,14,37^. Therefore, we investigated whether FBS also induces pY772-EphA2 in the NPC cells and observed that FBS induced the phosphorylation of EphA2 at Y772 and its downstream Shp2 and Erk-1/2 in the 5-8F and CNE2 NPC cells (Supplementary Fig. 2), indicating that growth factors in the serum induces ligand-independent pY772-EphA2 in the NPC cells, which is consistent with FBS inducing pS897-EphA2.

In summary, we found that ligand-independent pY772-EphA2 is responsible for EphA2-dependent NPC cell growth in vitro and in vivo by activating the Shp2/Erk-1/2 signaling pathway and pY772-EphA2 is a target of ALW-II-41-27 in the NPC cells (Fig. 7e). Our data suggest that pY772-EphA2 can serve as a therapeutic target in NPC and perhaps in other cancers.

## Materials and methods

### Antibodies, reagents, and plasmids

The antibodies against p-EphA2-Y772(Y772, #8244), p-EphA2 (S897, #6347), p-Shp2 (Y542, #3751), p-Shp2 (Y580, #5431), p-Gab1(Y627, #3233), p-ERK-1/2(#4370), p-AKT(S473, #4060), p-stat3 (S727, #9134), Gab1(#3232), AKT (#4691), and Stat3 (#4904) were purchased from CST (Cell Sigaling Technology). Antibodies against Shp2 (ab187040) and Grb2(ab32111) were purchased from Abcam. Antibodies against EphA2 (sc-924) and Erk-2 (sc-154) were purchased from Santa Cruz. Horseradish peroxidase (HRP)-conjugated goat anti-rabbit (A24531) and anti-mouse IgG antibodies (A24512), and Protein G/A-Sepharose™ 4B (#82085) were purchased from ThermoFisher Scientific. SHP099 (S8278) and U0126 (S1102) were purchased from Selleck Chemicals. ALW-II-41-27 (HY-18007) was purchased from MedChemExpress. CCK-8 (C0038) was purchased from Beyotime, Inc. EdU incorporation detection kit (C10310-1) was purchased from RiboBio, Inc.

Lentiviral vector GV341 expressing EphA2-Y772A cDNA was constructed by Genechem (Shanghai, China) and was confirmed by DNA sequencing. pBabepuro-EphA2 and -EphA2-S897A plasmids have been described previously by us^37^. pReceiver-M35-Shp2 and pReceiver-M13-ERK-2 expression plasmids were purchased from Genechem. pLKO.1 puro-Gab1-shRNA was a gift from Professor Hailing Cheng (Dalian Medical University, China) and pSIH-H1-puro-Shp2-shRNA was a gift from Professor Shan Gao (Suzhou Institute of System Medicine, Chinese Academy of Science, China). The target for Gab1 and Shp2 shRNA is 5′-GTTACGCAGTGGCCGTTTAAC-3′ and 5′-GGGAAAGAAGCAGAGAAATTATTAACTGA-3′, respectively.

### Cell lines and culture

5-8F and CNE2 NPC cell lines and 5-8F-shEphA2 and CNE2-shEphA2 NPC cell lines with stable knockdown of endogenous EphA2 by shRNA have been described previously by us^37^. Cells were cultured in RPMI-1640 medium supplemented with 10% FBS (Life Technologies) at 37 °C in 5% CO_2_. The cell lines were authenticated by short tandem repeat profiling prior to use and were routinely tested negative for mycoplasma contamination using 4,6-diamidino-2-phenylindole staining.

### IP and MS analysis

Total proteins were extracted from NPC 5-8F cells using NP-40 lysis buffer and 1.2 mg of total proteins were incubated with 30 μl Protein A/G- Sepharose™ 4B for 4 h at 4 °C followed by centrifugation for 5 min at 4 °C. The clarified supernatants were immunoprecipitated with 2 μg anti-EphA2 antibody and 30 μl Protein A/G-Sepharose 4B overnight at 4 °C, and centrifuged for 5 min at 4 °C. The beads were washed three times with PBS at 4 °C and boiled in 2× SDS-PAGE loading buffer for 5 min to elute protein complexes. The elutants were separated on SDS-PAGE gel followed by Coomassie brilliant blue G250 staining and then all protein bands were excised from the gels, in-gel trypsin digested, and subjected to NanoLC-MS/MS analysis with the Q Exactive hybrid quadrupole-Orbitrap mass spectrometer (ThermoFisher Scientific) to get MS/MS spectra as previously described^42^. Data of MS/MS were searched against Swiss-Prot database (*Homo sapiens*). Individual ions scores > 35 and unique peptide ≥ 1 indicate identity or extensive homology (*P* < 0.05) and were considered significant. All proteins deemed to be high confidence interactors of EphA2 were identified by at least two times of three replicates.

### Establishment of NPC cell lines expressing exogenous EphA2 or EphA2-Y772A using endogenous EphA2-knockdown cells

5-8F-shEphA2 and CNE2-shEphA2 NPC cells were infected with the lentiviral vector GV341 expressing EphA2-Y772A cDNA following the manufacturer’s instruction, or transfected with pBabepuro-EphA2 plasmid using Lipofectamine 2000 (ThermoFisher Scientific). Cells were selected using puromycin (ThermoFisher Scientific) for 2 weeks, and 5-8F and CNE2 cell lines with the stable expression of exogenous EphA2 or EphA2-Y772A and their control cell lines were obtained.

### Tumor formation assay in nude mice

Nude male mice (BALB/c nu/nu) that were 4 weeks old were obtained from the Laboratory Animal Center of Central South University and maintained in pathogen-free conditions. All animal experimental procedures were performed in accordance with the Guide for the Care and Use of Laboratory Animals of Xiangya Hospital, Central South University, with the approval of the Institutional Animal Ethics Committee.

The mice were randomly divided into the indicated groups before inoculation and 5 × 10^6^ cells resuspended in 200 μl of medium without serum were subcutaneously injected into the flanks of mice (*n* = 5 mice each). The mice were monitored daily for palpable tumor formation and tumor volume (in mm^3^) was measured by a vernier caliper every 3 days and calculated by using the modified ellipse formula (volume = length × width^2^/2). After 21 days, the mice were killed by cervical dislocation and their tumors were excised, weighed, and embedded in paraffin.

### Western blotting

Western blotting was performed as described previously by us^37^. Briefly, proteins were exacted from cells or tissues using RIPA lysis buffer. An equal amount of protein in each sample was subjected to SDS-PAGE separation, followed by blotting onto a PVDF membrane. After blocking, blots were incubated with primary antibody overnight at 4 °C, followed by incubation with HRP-conjugated secondary antibody for 1 h at room temperature. The signal was visualized with chemiluminescence detection reagent (Millipore).

### IP and immunoblotting (Co-IP)

Co-IP was performed to detect protein interaction. In brief, whole cell lysates were incubated with indicated antibodies and Protein G/A-Sepharose 4B overnight at 4 °C. After five times wash with RIPA buffer, beads were boiled in 2× SDS-PAGE loading buffer for 5 min to elute protein complexes, followed by SDS-PAGE separation and immunoblotting with specific antibodies.

### CCK-8 assay

Cell proliferation was measured using a CCK-8 kit as described previously by us^43^. The assay was performed three times in triplicate.

### Plate clone formation assay

Plate colony formation assay was performed to detect cell proliferation described previously by us^43^. The assay was performed three times in triplicate.

### EdU incorporation assay

EdU incorporation assay was performed to detect cell proliferation as described previously by us^43^. The assay was performed three times in triplicate.

### Soft agar colony formation assay

Soft agar colony formation assay was performed to detect cell anchorage-independent growth as described previously by us^43^. Cells were allowed to grow in the soft agar cultures for 12 days and colonies consisting of >50 cells were counted under the microscope. The assay was performed three times in triplicate.

### Immunohistochemistry

IHC analysis was performed on the formalin-fixed and paraffin-embedded xenograft tissue sections as described previously by us^37,43^. Briefly, tissue sections were incubated with anti-p-Shp2(Y542) antibody (YP5081, ImmunoWay Biotechnology; 1 : 200 dilution) or anti-p-Erk-1/2 antibody (1 : 400 dilution) overnight at 4 °C, and then incubated with biotinylated secondary antibody followed by avidin–biotin peroxidase complex (DAKO) at room temperature for 30 min. Finally, tissue sections were incubated with 3′, 3′-diaminobenzidine (Sigma-Aldrich) and counterstained with hematoxylin.

Staining intensity was categorized as follows: absent staining as 0, weak as 1, moderate as 2, and strong as 3. The percentage of stained cells (examined in at least 500 cells) was categorized as no staining = 0, <30% of stained cells = 1, 30~60% = 2, and >60% = 3. The staining score (ranging from 0 to 6) for each tissue was calculated by adding the area score and the intensity score. A combined staining score of ≤3 was considered to be low expression and >3 was considered to be high expression.

### Immunofluorescence

Immunofluorescent staining was performed as described previously by us^43^. Briefly, cells were plated into chamber slides (Millipore) and fixed in 4% paraformaldehyde, permeabilized, and incubated with mouse anti-EphA2 antibody (1 : 300 dilution) and rabbit anti-Shp2 antibody (1 : 100 dilution), followed by incubation with DyLight® 488 anti-mouse IgG (DI-2788, Vector Laboratories) and DyLight® 594 anti-Rabbit IgG (DI-1794, Vector Laboratories). Images were captured using an inverted confocal fluorescent microscope (LEICA TCS SP8). Nuclei were counterstained with 4′,6-diamidino-2-phenylindole (DAPI).

### Statistical analysis

Statistical analysis was performed using IBM SPSS statistical software package 22. Data are presented as means ± SD. Qualitative variables were compared by the Student’s *t*-test or *χ*^2^-test. All statistical tests were two-sided and *p*-values < 0.05 were considered statistically significant.

## Supplementary information

Figure S1

Figure S2

Supplementary Figure legends
