# Supplementary figures and images for: Y772 phosphorylation of EphA2 is responsible for EphA2-dependent NPC nasopharyngeal carcinoma growth by Shp2/Erk-1/2 signaling pathway
Source: Cell Death Dis. 2020 Aug 27;11(8):709. doi: 10.1038/s41419-020-02831-0 (PMC7449971; doi:10.1038/s41419-020-02831-0)

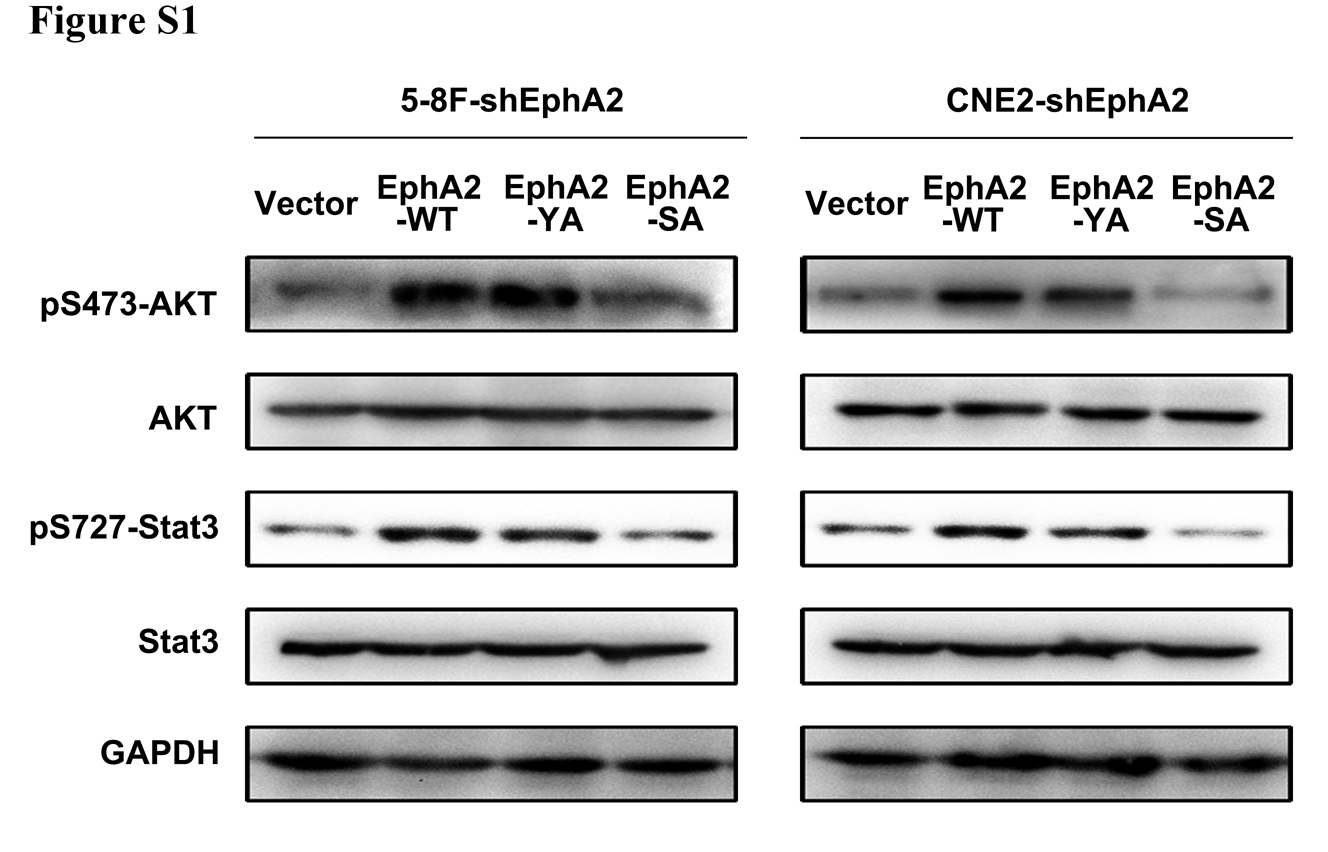

Supplement: Supplementary file 1 — Figure S1 [file 41419_2020_2831_MOESM1_ESM.tif]

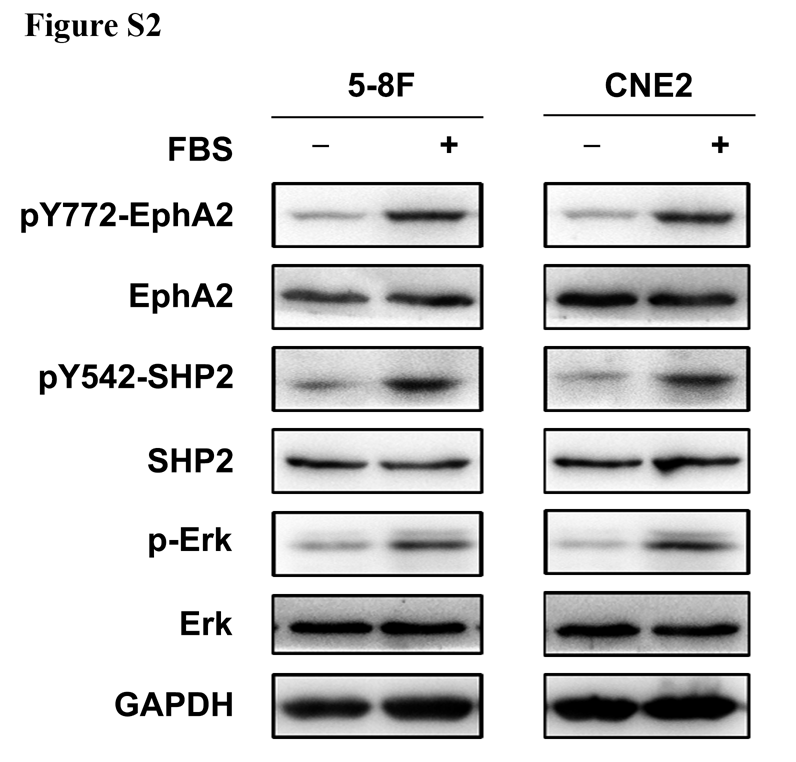

Supplement: Supplementary file 2 — Figure S2 [file 41419_2020_2831_MOESM2_ESM.tif]
