# Supplementary material for: Y772 phosphorylation of EphA2 is responsible for EphA2-dependent NPC nasopharyngeal carcinoma growth by Shp2/Erk-1/2 signaling pathway
Source: Cell Death Dis. 2020 Aug 27;11(8):709. doi: 10.1038/s41419-020-02831-0 (PMC7449971; doi:10.1038/s41419-020-02831-0)
Supplement: Supplementary file 3 — Supplementary Figure legends [file 41419_2020_2831_MOESM3_ESM.doc]

**Supplemental Figure 1. The effect of pY772-EphA2 and EphA2-S897A on AKT/Stat3 signaling activity in the NPC cells.** Western blot showing the levels of p-AKT and p-Stat3 in the NPC cells expressing EphA2-WT, EphA2-YA or EphA2-SA and their control cells.

**Supplemental Figure 2**. **Fetal bovine serum (FBS) induces ligand-independent phosphorylation of EphA2 at Y772 in the NPC cells.** Western blot showing the levels of pY772-EphA2, p-Shp2 and p-Erk-1/2 in the 5-8F and CNE2 NPC cells stimulated with FBS for 30min after serum-starved for 12h.
